# Evaluation of Texture Analysis for the Differential Diagnosis of Mass-Forming Pancreatitis From Pancreatic Ductal Adenocarcinoma on Contrast-Enhanced CT Images

**DOI:** 10.3389/fonc.2019.01171

**Published:** 2019-11-05

**Authors:** Shuai Ren, Jingjing Zhang, Jingya Chen, Wenjing Cui, Rui Zhao, Wenli Qiu, Shaofeng Duan, Rong Chen, Xiao Chen, Zhongqiu Wang

**Affiliations:** ^1^Department of Radiology, Affiliated Hospital of Nanjing University of Chinese Medicine, Jiangsu Province Hospital of Chinese Medicine, Nanjing, China; ^2^GE Healthcare China, Shanghai, China; ^3^Department of Diagnostic Radiology and Nuclear Medicine, University of Maryland School of Medicine, Baltimore, MD, United States

**Keywords:** pancreatic ductal adenocarcinoma, chronic pancreatitis, computed tomography, texture analysis, machine learning

## Abstract

**Purpose:** To investigate the potential of computed tomography (CT) imaging features and texture analysis to differentiate between mass-forming pancreatitis (MFP) and pancreatic ductal adenocarcinoma (PDAC).

**Materials and Methods:** Thirty patients with pathologically proved MFP and 79 patients with PDAC were included in this study. Clinical data and CT imaging features of the two lesions were evaluated. Texture features were extracted from arterial and portal phase CT images using commercially available software (AnalysisKit). Multivariate logistic regression analyses were used to identify relevant CT imaging and texture parameters to discriminate MFP from PDAC. Receiver operating characteristic curves were performed to determine the diagnostic performance of predictions.

**Results:** MFP showed a larger size compared to PDAC (*p* = 0.009). Cystic degeneration, pancreatic ductal dilatation, vascular invasion, and pancreatic sinistral portal hypertension were more frequent and duct penetrating sign was less frequent in PDAC compared to MFP. Arterial CT attenuation, arterial, and portal enhancement ratios of MFP were higher than PDAC (*p* < 0.05). In multivariate analysis, arterial CT attenuation and pancreatic duct penetrating sign were independent predictors. Texture features in arterial phase including SurfaceArea, Percentile40, InverseDifferenceMoment_angle90_offset4, LongRunEmphasis_angle45_offset4, and uniformity were independent predictors. Texture features in portal phase including LongRunEmphasis_angle135_offset7, VoxelValueSum, LongRunEmphasis_angle135_offset4, and GLCMEntropy_angle45_offset1 were independent predictors. Areas under the curve of imaging feature-based, texture feature-based in arterial and portal phases, and the combined models were 0.84, 0.96, 0.93, and 0.98, respectively.

**Conclusions:** CT texture analysis demonstrates great potential to differentiate MFP from PDAC.

## Introduction

Pancreatic ductal adenocarcinoma (PDAC) is a deadly disease with a grim overall prognosis; its mortality is almost equal to its morbidity (1). The 5-year survival rate for PDAC patients was 7.8% between 2006 and 2012, compared to a 66.9% 5-year survival rate for cancers at all sites (2). Surgical resection is regarded as the only curative treatment option, but most patients are asymptomatic and diagnosed at an advanced stage when the optimal time for surgical resection has passed (1). Therefore, effective and non-invasive screening methods to detect PDAC at an early stage are of utmost importance. This may help to increase the survival rate by providing a chance for early surgical treatment and adjuvant intervention (3, 4).

Mass-forming pancreatitis (MFP) encompasses a gradual form of the ordinary chronic pancreatitis or a specific etiology such as focal type of autoimmune pancreatitis (5, 6), which must be differentiated accurately from PDAC due to their similar presentations of abdominal pain, weight loss, pancreatic insufficiency, and overlapping radiologic features (7–11). Resectional surgery is radical for MFP and may result in pancreatic insufficiency. On the other hand, drainage operations are ineffective for PDAC. The incidence of PDAC in the MFP population is estimated to be 1–6% (12–15). An estimated 5–10% of patients who undergo pancreaticoduodenectomy for suspected pancreatic malignancy are found to have benign lesions on surgical pathologic examination (12, 13). Thus, accurate preoperative differentiation between MFP and PDAC is clinically important for deciding whether or not to perform resection (12). With the application of endoscopic ultrasound fine-needle aspiration biopsy (EUS-FNA), cytopathological evidence can be acquired accurately from pancreatic masses with a reported sensitivity of 79–98%, specificity of 71–100%, and accuracy of 82–98% (5, 16, 17). However, even EUS-guided biopsies show false negative diagnosis of 12–14%, which may result in delayed patient care (5). Therefore, non-invasive imaging is crucial in the differential diagnosis and treatment strategy planning.

Several previous studies have proved that dual-energy CT, perfusion CT, and diffusion-weighted magnetic resonance imaging can be utilized for discriminating MFP from PDAC (7–10, 13, 18). With the application of extracting, analyzing, and interpreting quantitative imaging features, texture analysis has recently been applied to cancer diagnosis, treatment response, and prognosis evaluation (19). However, no studies have investigated the potential of texture analysis in differential diagnosis between the two lesions. The aim of our study was to investigate the diagnostic performance of CT imaging features and texture analysis in the differentiation between the two lesions.

## Materials and Methods

Patient data confidentiality was protected in accordance with the Declaration of Helsinki principles. The protocol was approved by the institutional review board of the Affiliated Hospital of Nanjing University of Chinese Medicine. Written informed consent was waived because it is a retrospective study.

### Patient Selection

**Figure 1:**
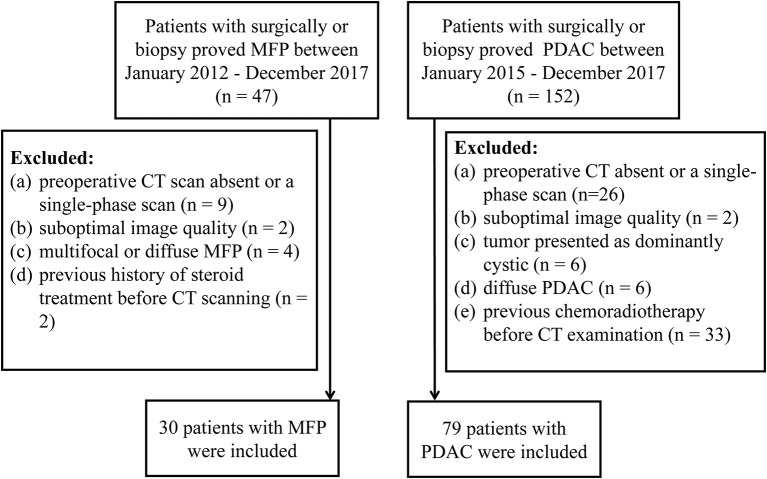
Flow diagram of patients' selection.

From January 2012 to December 2017, 47 consecutive patients with surgically- (*n* = 29) or biopsy- (*n* = 18) proved MFP were identified from our medical records. Inclusion criteria were: (1) contrast-enhanced CT performed <30 days prior to resection or biopsy; (2) clinical characteristics available. The exclusion criteria were: (1) preoperative CT scan absent or only single-phase obtained (*n* = 9); or (2) suboptimal image quality (*n* = 2); (3) multifocal or diffuse MFP (*n* = 4); (4) history of steroid treatment before CT scan (*n* = 2). Ultimately, a total of 30 patients (25 males and 5 females; mean age 61.47 ± 12.43 years; age range 43–74 years) were included in our study (Figure 1).

Similarly, from January 2015 to December 2017,152 consecutive patients with surgically- (*n* = 130) or biopsy- (*n* = 22) proved PDAC were identified from our medical records. Inclusion criteria were: (1) contrast-enhanced CT performed <30 days prior to resection or biopsy; (2) clinical characteristics available. The exclusion criteria were: (1) preoperative CT scan absent or only single-phase scan obtained (*n* = 26); or (2) suboptimal image quality (*n* = 2); or (3) tumor presented as dominantly cystic (*n* = 6); (4) diffuse PDAC (*n* = 6); (5) previous chemoradiotherapy before CT examination (*n* = 33). Ultimately, a total of 79 patients (56 males and 23 females; mean age 65.18 ± 8.60 years; age range, 61–84 years) were included in our study (Figure 1).

### CT Imaging

Multi-detector CT systems, including Philips Brilliance 64 (Philips Healthcare, DA Best, the Netherlands) and Optima 670 (GE Healthcare, Tokyo, Japan), were used for CT scanning following a standardized protocol. Three-phase examinations consisted of unenhanced, arterial, and portal phases were performed on all patients. CT scanning parameters were as follows: tube voltage, 120 kVp; current, 200–400 mAs; pitch, 1.375; rotation speed, 0.75 s; slice thickness, 3.0 mm; slice interval, 3.0 mm; and a reconstruction interval of 1.25 mm. Following unenhanced imaging, non-ionic contrast media Ultravist 300 (Bayer Schering Pharma AG, Berlin, Germany) was administrated intravenously (1.2–1.5 ml/kg) at a rate of 3.0 ml/s followed by 40 ml saline solution, using a power injector (Ulrich Medical, Ulm, Germany). Arterial and portal phase scanning were obtained at 35 and 60 s.

### Image Analysis

CT images were evaluated by two independent blinded abdominal radiologists (SR and JZ). Disparities in image analysis were resolved by discussion with a senior radiologist (JC) until consensus was reached. The following imaging features were evaluated: tumor location, shape, margin, cystic degeneration, calcification, enhancement pattern, degree and shape of pancreatic ductal dilatation, pancreatic duct penetrating sign, vascular invasion, pancreatic sinistral portal hypertension, peripancreatic exudation, and lymph nodes enlargement. A smooth and clearly visible margin was considered to be well-defined, while spiculation or infiltration on >90° of tumor perimeter was considered to be ill-defined (20). Tumor thrombus, vessel occlusion, stenosis or contour deformity indicated vascular invasion (21). An enhancing solid portion of <50% of the tumor was regarded as cystic degeneration (22). Unenhanced images was used to identify calcifications. The senior radiologist (JC) measured size (cm) and CT attenuation (HU) of lesions and adjacent parenchyma three times with region of interest (ROI) delineating. ROIs encompassed the solid components, avoiding intratumoral calcification, and cystic degeneration. Arterial and portal enhancement ratios were calculated by dividing CT attenuation (HU) of the tumor by that of the pancreatic parenchyma in arterial and portal phases, respectively (17).

### Texture Analysis

**Figure 2:**
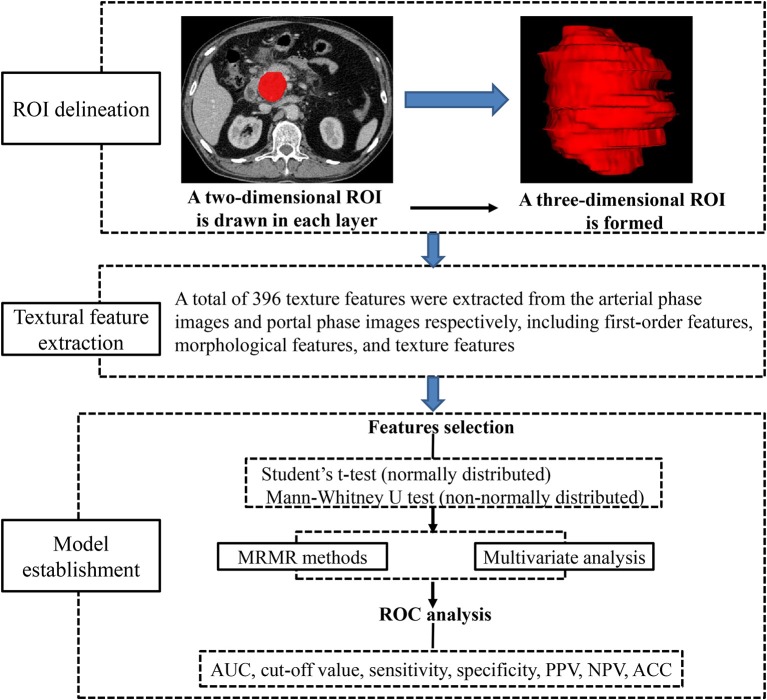
Flow diagram of image processing and texture features calculation.

Preoperative images obtained in arterial and portal phases were exported in DICOM format from medical database to ITK-SNAP for ROI delineation. ROIs were manually drawn along the margin of the tumor in every visualized tumor image in consensus by two radiologists (SR and JC), avoiding peripheral fat, artifacts, and blood vessels in order to eliminate non-tumor tissue effect. All DICOM images and their corresponding ROIs were individually transferred to AnalysisKit software (Version V3.0.0.R, GE Healthcare) for texture features extraction. Image processing and texture feature analysis are demonstrated in Figure 2. As referring to Image Biomarker Standardization Initiative (ISBI), three categories of radiomic features were calculated. (1) first-order features, which describe the distribution of voxel intensities within the ROI. (2) Morphological features, which describe geometric aspects of a ROI, such as area and volume. (3) Texture features, which describe high-order gray information within the ROI, they are calculated based on three matrices, the gray-level co-occurrence matrix (GLCM), a matrix that expresses how combinations of discretized gray levels of neighboring pixels, or voxels in a 3D volume, are distributed along one of the image directions, the run length matrix (RLM), assesses the length of a consecutive sequence of pixels or voxels with the same gray level along one direction, the gray level size zone matrix (GLSZM), counts the number of groups (or zones) of linked voxels. A total of 396 texture features were extracted from each image in our study.

### Statistical Analysis

Statistical analysis was performed with R software, version 3.5.3 (https://www.r-project.org). Conventional contrast-enhanced CT imaging features were compared between MFP and PDAC using Chi-square or Fisher's exact test for categorical variables and the Student's *t*-test or Mann-Whitney *U*-test for continuous variables. Minimum redundancy maximum relevance (MRMR) was performed for radiomic feature selection to reduce the redundancy and unnecessary complexity of the computation and modeling (23). A stepwise-backward multivariate logistic regression was used to identify relevant CT imaging and texture features and construct radiomic models to discriminate MFP from PDAC. Receiver operating characteristic (ROC) curve analysis was used to validate the performance of predicting models by providing area under the curve (AUC), sensitivity, specificity, accuracy, positive predictive value (PPV) and negative predictive value (NPV). *P* < 0.05 was considered statistically significant.

## Results

### Patients' Clinical Characteristics

The clinical characteristics of 30 MFP and 79 PDAC patients are summarized in Table 1. No significant differences were found in age, sex or clinical symptoms between the two groups with the exception of a history of pancreatitis, which was more common in MFP than PDAC (73.1 vs. 14.6%, *p* < 0.001).

**Table 1:** Clinical data of patients with MFP and PDAC.

**Variables**	**MFP (*n* = 30)**	**PDAC (*n* = 79)**	***P*-value**
Age (years)	61.47 ± 12.43	65.18 ± 8.60	0.140
Gender			0.184
Male	25 (83.33%)	56 (70.89%)	
Female	5 (16.67%)	23 (29.11%)	
Clinical symptoms			
A history of pancreatitis	19 (73.1%)	12 (14.6%)	<0.001
Abdominal pain	25 (83.33%)	62 (78.48%)	0.573
Yellow urine or icterus	8 (26.67%)	27 (34.18%)	0.453
Nausea and vomiting	5 (16.67%)	11 (13.92%)	0.953
Weight loss	2 (6.67%)	13 (16.46%)	0.311
Fever	3 (10.00%)	2 (2.53%)	0.249

### Comparison of CT Imaging Features Between MFP and PDAC

**Figure 3:**
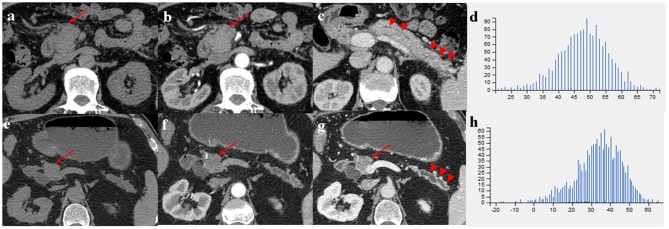
**(a–c)** A 66-year-old man with MFP, CT imaging showed a pancreatic head tumor (arrows) with slight duct dilatation and slight tail enlargement (arrowheads); **(e–g)** A 83-year-old man with PDAC, CT imaging showed a pancreatic head tumor (arrows) with severe duct dilatation and obvious tail atrophy (arrowheads). **(d,h)** Histograms of texture parameters of the two lesions showed a marked difference.

**Figure 4:**
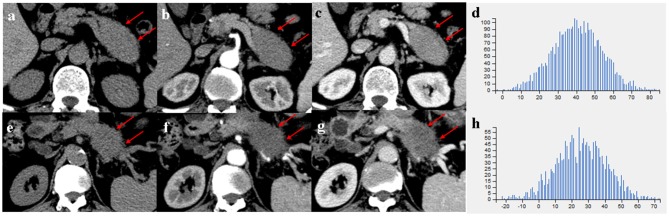
**(a–c)** A 66-year-old man with MFP, CT imaging showed a pancreatic body tumor (arrows) with a gradual enhancement pattern, the margin of which was well-defined; **(e–g)** A 68-year-old man with PDAC, CT imaging showed a pancreatic body tumor (arrows) with a hypovascular pattern, the margin of which was ill-defined. **(d,h)** Histograms of texture parameters of the two lesions showed a marked difference.

Comparison of CT imaging features between MFP and PDAC is summarized in Table 2. No statistically significant differences with respect to tumor location, shape, margin, calcification, peripancreatic exudation, or lymph nodes enlargement were observed between MFP and PDAC patients. Pancreatic ductal dilatation was more frequent in PDAC than MFP [59.5% (47 of 79) vs. 36.7% (11 of 30), *p* = 0.033]. However, no statistically significant difference with respect to degree of pancreatic ductal dilatation was observed between the two groups. In those lesions with duct dilatation, uniformity of dilatation was more common in PDAC than MFP [83.0% (39 of 47) vs. 36.4% (4 of 11)] and beaded dilatation was more common in MFP than PDAC [63.6% (7 of 11) vs. 17.0% (8 of 47)] with a *p*-value of 0.005. Pancreatic duct penetrating sign was more common in MFP compared to PDAC [66.7% (20 of 30) vs. 19.0% (15 of 79), *p* < 0.001]. Moreover, cystic degeneration, vascular invasion, and pancreatic sinistral portal hypertension were more frequent in PDAC than MFP (all *p* < 0.05). Cystic degeneration was found in 46.8% (37/79) of PDAC, vascular invasion was found in 53.2% (42/79) of PDAC, and pancreatic sinistral portal hypertension was found in 39.2% (31/79) of PDAC, while only 25.8% (8/30), 16.7% (5/30), and 13.3% (4/30), respectively, of MFP exhibited such imaging features. Figures 3, 4 show representative cases of MFP (Figures 3a–c, 4a–c) and PDAC (Figures 3e–g, 4e–g) tumors located at pancreatic head and body, respectively.

**Table 2:** CT imaging features of patients with MFP and PDAC.

**CT findings**	**MFP (*n* = 30)**	**PDAC (*n* = 79)**	***p*-value**
Location			0.150
Head and neck	24 (80%)	52 (65.8%)	
Body and tail	6 (20%)	27 (34.2%)	
Shape (oval/lobulation/ irregular)	20/3/7	62/5/12	0.443
Margin			0.707
Well-defined	2 (6.7%)	9 (11.4%)	
Ill-defined	28 (93.3%)	70 (88.6%)	
Cystic degeneration	8 (25.8%)	37 (46.8%)	0.044
Calcification	6 (20%)	6 (7.6%)	0.065
Enhancement pattern			0.140
Homogeneous	21 (70%)	43 (54.4%)	
Heterogeneous	9 (30%)	36 (45.6%)	
Pancreatic ductal dilatation	11 (36.7%)	47 (59.5%)	0.033
Degree (low to moderate/severe)	9/2	34/13	0.792
Shape (Uniformity/Beaded expansion)	4/7	39/8	0.005
Pancreatic duct penetrating sign	20 (66.7%)	15 (19%)	<0.001
Vascular invasion	5 (16.7%)	42 (53.2%)	0.001
Pancreatic sinistral portal hypertension	4 (13.3%)	31 (39.2%)	0.010
Peripancreatic exudation	11 (36.7%)	18 (22.8%)	0.143
Lymph nodes enlargement	12 (40.0%)	42 (53.2%)	0.220
Size (cm)	4.05 ± 1.02	3.41 ± 1.16	0.009
Arterial CT attenuation (HU)	60.57 ± 11.24	51.82 ± 11.36	<0.001
Portal CT attenuation (HU)	75.17 ± 11.04	71.53 ± 13.94	0.203
Arterial enhancement ratio	0.89 ± 0.20	0.66 ± 0.15	<0.001
Portal enhancement ratio	0.89 ± 0.19	0.77 ± 0.17	0.004

*HU, Hounsfield unit*.

**Figure 5:**
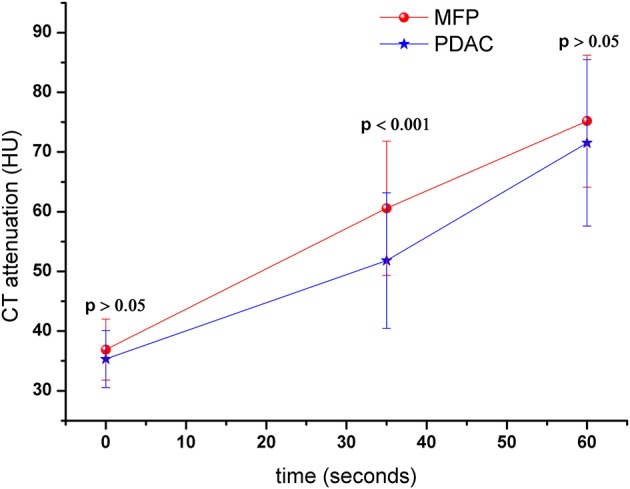
Dynamic contrast-enhanced curves of patients with MFP and PDAC. CT attenuation of MFP and PDAC was 41.52 ± 5.79 and 42.00 ± 5.37 Hounsfield units in unenhanced phase, respectively. CT attenuation of MFP was higher than PDAC in arterial phase (*p* < 0.001).

**Figure 6:**
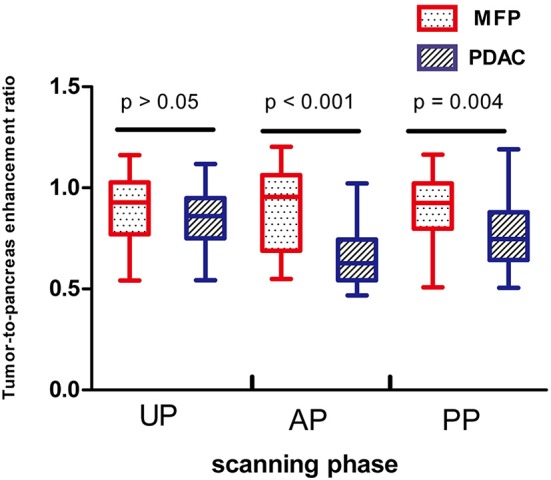
Box-and-whisker plots of enhancement ratios of patients with MFP and PDAC in unenhanced, arterial, and portal phases. Enhancement ratios of MFP were higher than PDAC in arterial phase (AP, *p* < 0.001) and portal phase (PP, *p* = 0.004).

Tumor size was larger in MFP than PDAC (4.05 cm ± 1.02 vs. 3.41 cm ± 1.16, *p* = 0.009). Arterial CT attenuation of MFP was significantly higher than PDAC (59.24 ± 13.11 HU vs. 44.36 ± 8.66 HU, *p* < 0.001), while no significant difference was observed between MFP and PDAC with respect to unenhanced or portal CT attenuation (*p* > 0.05; Figure 5). Arterial and portal enhancement ratios of MFP were significantly higher than PDAC (*p* < 0.001, *p* = 0.004; Figure 6).

**Figure 7:**
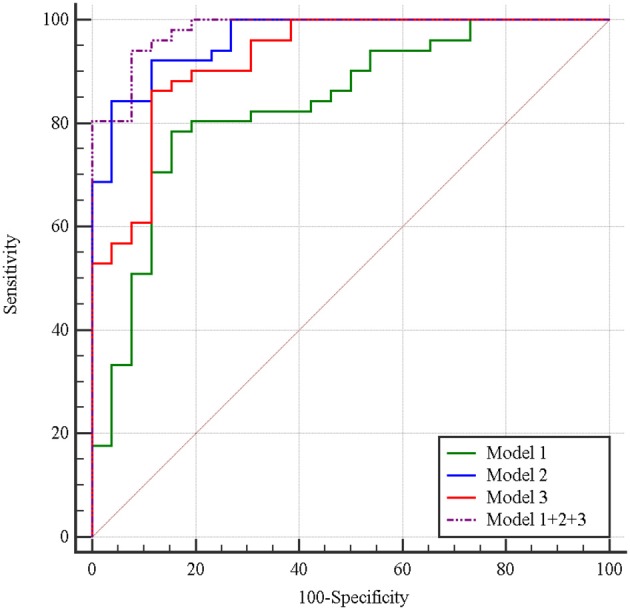
Receiver operating characteristic curves of imaging feature-based (Model 1), texture feature-based models in arterial phase (Model 2), and portal phase (Model 3), and the combined model (Model 1 + 2 + 3) in differentiating MFP from PDAC. The areas under the curve were 0.84, 0.96, 0.93, and 0.98, respectively.

Multivariate logistic regression was performed to ascertain relevant CT imaging features in differentiating the two lesions. Arterial CT attenuation and pancreatic duct penetrating sign were independent predictors of CT imaging features in differentiating the two lesions (Table 3). The diagnostic performance of each imaging feature is demonstrated in Supplementary Table 1. ROC curve was adopted to determine the diagnostic performance of imaging feature-based model (Model 1) in differentiating MFP from PDAC (Figure 7). Parameters of Model 1 including AUC, cut-off value, diagnostic sensitivity (%), specificity (%), accuracy (%), PPV (%), and NPV (%) were 0.84, 0.787, 77, 86, 72, 91, and 57%, respectively (Table 4).

**Table 3:** Multivariate analyses of CT imaging and texture parameters in differentiating MFP from PDAC.

	**Characteristics**	**OR**	**95% (CI)**	***P*-value**
CT features				
	Arterial CT attenuation	0.943	0.911–0.976	0.001
	Pancreatic duct penetrating sign	0.119	0.043–0.333	<0.001
Texture features				
AP				
	Surface Area	0.282	0.095–0.672	0.008
	Percentile40	0.042	0.005–0.201	<0.001
	InverseDifferenceMoment_angle90_offset4	0.129	0.024–0.486	0.006
	LongRunEmphasis_angle45_offset4	0.326	0.095–0.868	0.041
	uniformity	6.722	1.124–40.268	0.029
PP				
	LongRunEmphasis_angle135_offset7	0.394	0.104–1.274	0.060
	VoxelValueSum	0.253	0.097–0.546	0.001
	LongRunEmphasis_angle135_offset4	0.529	0.209–1.244	0.069
	GLCMEntropy_angle45_offset1	2.169	0.946–6.773	0.007

*OR, odds ratio; CI, confidence interval; AP, arterial phase; PP, portal phase*.

**Table 4:** Diagnostic performance of imaging feature-based (Model 1), texture feature-based models in arterial phase (Model 2), and portal phase (Model 3), and the combined model (Model 1 + 2 + 3) for differentiating MFP from PDAC.

**Model**	**AUC** **(95% CI)**	**Cut-off value** **(SEN%, SPE%)**	**PPV** **(%)**	**NPV** **(%)**	**ACC** **(%)**
Model 1	0.84 (0.75–0.92)	0.787 (77, 86)	91	57	72
Model 2	0.96 (0.93–1.0)	1.502 (96, 83)	74	98	87
Model 3	0.93 (0.87–0.98)	0.347 (77, 94)	87	89	89
Model 1 + 2 + 3	0.98 (0.92–1.0)	0.598 (94, 92)	96	89	94

*AUC, area under the curve; SEN, sensitivity; SPE, specificity; PPV, positive predictive value; NPV, negative predictive value; CI, confidence interval*.

### Comparison of CT Texture Analysis Between MFP and PDAC

A total of 396 texture features were extracted from each image in arterial or portal phase. Multivariate logistic regression was performed to ascertain relevant CT texture features in differentiating the two lesions. SurfaceArea, Percentile40, InverseDifferenceMoment_angle90_offset4, LongRunEmphasis_angle45_offset4, and uniformity were independent predictors of arterial texture parameters in differentiating the two lesions (Table 3). LongRunEmphasis_angle135_offset7, VoxelValueSum, LongRunEmphasis_angle135_offset4, and GLCMEntropy_angle45_offset1 were independent predictors of portal texture parameters in differentiating the two lesions (Table 3).The diagnostic performance of each texture feature in arterial and portal phases is shown in Supplementary Table 1. ROC curves were adopted to determine the diagnostic performance of arterial texture feature-based model (Model 2) and portal texture feature-based model (Model 3) in differentiating MFP from PDAC (Figure 7). Parameters of Model 2 including AUC, cut-off value, sensitivity (%), specificity (%), accuracy (%), PPV (%), and NPV (%) were 0.96, 1.502, 96, 83, 87, 74, and 98%, respectively (Table 4). Parameters of Model 3 including AUC, cut-off value, diagnostic sensitivity (%), specificity (%), accuracy (%), PPV (%), and NPV (%) were 0.93, 0.347, 77, 94, 89, 87, and 89%, respectively (Table 4).

Subsequently, the diagnostic ability of the combined model based on imaging features and texture features (Model 1 + 2 + 3) was also evaluated via ROC curve analysis (Figure 7). The AUC was 0.98 with 94% sensitivity, 92% specificity, 94% accuracy, 96% PPV, and 89% NPV for discriminating MFP from PDAC (Table 4).

Finally, we randomly selected 40 samples for internal validation, including 10 MFP (33.3%), and 30 PDAC tumors (38.0%). The AUCs of texture feature-based models in arterial and portal phases were 0.90 (90% sensitivity, 81% specificity, 64% PPV, 96% NPV, and 75% accuracy) and 0.87 (66% sensitivity, 96% specificity, 52% PPV, 98% NPV, and 74% accuracy), respectively. The verification results are similar to previous studies, which prove that the model is comparatively reproducible and stable.

## Discussion

Chronic pancreatitis and PDAC are commonly seen in clinical practice, but only a small portion of chronic pancreatitis presents as MFP. The overlap in clinical and radiologic features of the two lesions makes their early preoperative differential diagnosis difficult (7–11, 24–26). Misdiagnosis of MFP as PDAC may result in unnecessary interventional procedures, and misdiagnosis of PDAC as MFP may result in delayed surgical treatment (10). In the present study, we evaluated the role of CT imaging and texture analysis in differentiating MFP from PDAC. Our data indicate that CT texture analysis demonstrates great potential in differentiating MFP from PDAC.

Treatment strategies and management are geared to pancreatic tumor type. Early preoperative differentiation between MFP and PDAC would be especially useful for treatment planning. Although a fine-needle biopsy is an accurate method in tumor identification, imaging plays a crucial role in estimating the aggressiveness of the tumor and facilitating in treatment approaches planning. Previous CT or MRI studies indicate that internal cystic or necrotic portion, peripancreatic fat infiltration, vascular invasion, uniformity of pancreatic duct expansion, and lymph nodes enlargement are more frequent in PDAC, while beaded pancreatic duct expansion and duct penetrating sign are more frequent in MFP (10, 18, 24). Our data is in consistent with the findings, and in addition we found that pancreatic sinistral portal hypertension was more common in PDAC than MFP. We also evaluated the value of CT attenuation and enhancement ratio in differentiating MFP from PDAC. We found that arterial CT attenuation, arterial and portal enhancement ratios were significantly higher in MFP than PDAC. In multivariate analyses, arterial CT attenuation and pancreatic duct penetrating sign were independent predictors in differentiating MFP from PDAC.

CT and MRI imaging findings provide mostly qualitative evaluation. The use of quantitative parameters could enhance diagnosis. Texture analysis is an emerging imaging-based post-processing method that allows for quantification of tissue heterogeneity (27). There has been a surge in recent years in the research application of CT texture analysis in tumor identification, staging, and therapy response assessment (19, 23, 27, 28). However, no studies have demonstrated the value of CT texture analysis in differentiating MFP from PDAC. We are the first to use CT texture analysis to differentiate MFP from PDAC. Our data show that SurfaceArea, Percentile40, InverseDifferenceMoment_angle90_offset4, LongRunEmphasis_angle45_offset4, and uniformity on arterial phase images and LongRunEmphasis_angle135_offset7, VoxelValueSum, LongRunEmphasis_angle135_offset4, and GLCMEntropy_angle45_offset1 on portal phase images are independent predictors in discriminating MFP from PDAC. Moreover, our data indicate that CT imaging features can obtain good specificity but poor sensitivity in differentiating the two lesions. However, texture analyses in arterial phase can obtain high pooled sensitivity and specificity. The combined model based on imaging features and texture features reveal high pooled sensitivity of 94%, specificity of 92%, accuracy of 94, 96% PPV, and 89% NPV. Texture analyses can effectively improve the efficacy of contrast-enhanced CT for differentiating MFP from PDAC.

We acknowledge the following limitations of our study. First, only patients with pathologically proved MFP or PDAC were included in our study, which may result in a selection bias due to the lower incidence of MFP than PDAC. However, this step is an essential inclusion criterion for the aim of validating the correlation between CT texture analyses and tumor types. Second, inter-observer variability for tumor segmentation could not be obtained due to the consensus review by radiologists. Third, the enrolled number of patients is relatively small for CT texture analysis. There are two main reasons for the relatively small sample size: (1) MFP is a special type of chronic pancreatitis, which accounts for 10–30% of chronic pancreatitis (11). It is relatively difficult to collect enough patients in a short time period. (2) To meet the requirement of high consistency of scanning equipment and parameter and to guarantee the accuracy of texture analysis (29), some cases should be excluded from the cohort. But since the enrolled patients were not sufficient, all cases were included, and an internal validation was adopted to verify the results in order to guarantee the accuracy of the test set as best as the existing conditions permit. A multicenter program to include more MFP patients may be needed, and an external validation to confirm the potential value of CT texture analyses in discriminating MFP from PDAC may also be needed.

In conclusion, we found that multiple CT imaging and texture parameters are significantly different between MFP and PDAC groups. Larger-cohort studies, preferably multicenter, to confirm the potential value of CT texture analyses in differentiating MFP from PDAC are in order.

## Data Availability Statement

All datasets generated for this study are included in the article/Supplementary Material.

## Author Contributions

SR, XC, and ZW designed the study. JZ, JC, WC, WQ, and RZ conducted the experiments. JC, SD, and RC analyzed the data. SR wrote the draft. All authors read and approved the final manuscript.

### Conflict of Interest

SD was employed by the company GE healthcare. The remaining authors declare that the research was conducted in the absence of any commercial or financial relationships that could be construed as a potential conflict of interest.
