# Supplementary material for: Evaluation of Texture Analysis for the Differential Diagnosis of Mass-Forming Pancreatitis From Pancreatic Ductal Adenocarcinoma on Contrast-Enhanced CT Images
Source: Front Oncol. 2019 Nov 5;9:1171. doi: 10.3389/fonc.2019.01171 (PMC6848378; doi:10.3389/fonc.2019.01171)
Supplement: Supplementary file 1 [file Table_1.docx]

| Variables | AUC  (95%CI) | Cut-off value  (SEN%, SPE%) | PPV  (%) | NPV  (%) | ACC  (%) |
| --- | --- | --- | --- | --- | --- |
| CT features |  |  |  |  |  |
| Arterial CT attenuation | 0.73 (0.61-0.85) | 63.5 (57, 87) | 63 | 84 | 57 |
| Pancreatic duct penetrating sign | 0.72 (0.61-0.84) | 0.5 (67, 81) | 57 | 86 | 67 |
| Texture parameters in AP |  |  |  |  |  |
| SurfaceArea | 0.87 (0.78-0.96) | 1718.70 (85, 81) | 90 | 72 | 84 |
| Percentile40 | 0.83 (0.73-0.93) | 46.13 (74, 81) | 89 | 60 | 76 |
| InverseDifferenceMoment_angle90_offset4 | 0.83 (0.73-0.93) | 0.03 (74, 81) | 89 | 60 | 76 |
| LongRunEmphasis_angle45_offset4 | 0.74(0.62-0.85) | 1.08 (55, 85) | 88 | 48 | 65 |
| uniformity | 0.79 (0.67-0.92) | 0.77 (94, 65) | 85 | 85 | 85 |
| Texture parameters in PP |  |  |  |  |  |
| LongRunEmphasis_angle135_offset7 | 0.81 (0.71-0.91) | 1.36(100, 50) | 80 | 100 | 84 |
| VoxelValueSum | 0.82 (0.72-0.93) | 200743 (79, 81) | 89 | 66 | 80 |
| LongRunEmphasis_angle135_offset4 | 0.77 (0.66-0.88) | 1.07 (53, 96) | 97 | 50 | 67 |
| GLCMEntropy_angle45_offset1 | 0.75 (0.62-0.83) | 10.68 (83, 69) | 85 | 67 | 78 |

Supplementary Table 1 Diagnostic performance of CT imaging features and texture parameters in differentiating MFP from PDAC

AP, arterial phase; PP, portal phase; AUC, area under the curve; CI, confidence interval; SEN, sensitivity; SPE, specificity; PPV, positive predictive value; NPV, negative predictive value; ACC, accuracy
